# Lifestyle and Income-related Inequality in Health in South Africa

**DOI:** 10.1186/s12939-017-0598-7

**Published:** 2017-06-19

**Authors:** Alfred Kechia Mukong, Corne Van Walbeek, Hana Ross

**Affiliations:** Economics of Tobacco Control Project, South African Labour and Development Research Unit (SAL-DRU), School of Economics, University of Cape Town, Rondebosch, South Africa

**Keywords:** Alcohol and smoking, Health inequality, Concentration index, I12, I14, C19

## Abstract

**Background:**

Many low- and middle-income countries are experiencing an epidemiological transition from communicable to non-communicable diseases. This has negative consequences for their human capital development, and imposes a growing economic burden on their societies. While the prevalence of such diseases varies with socioeconomic status, the inequalities can be exacerbated by adopted lifestyles of individuals. Evidence suggests that lifestyle factors may explain the income-related inequality in self-reported health. Self-reported health is a subjective evaluation of people’s general health status rather than an objective measure of lifestyle-related ill-health.

**Method:**

The objective of this paper is to expand the literature by examining the contribution of smoking and alcohol consumption to health inequalities, incorporating more objective measures of health, that are directly associated with these lifestyle practices. We used the National Income Dynamic Study panel data for South Africa. The corrected concentration index is used to measure inequalities in health outcomes. We use a decomposition technique to identify the contribution of smoking and alcohol use to inequalities in health.

**Results:**

We find significant smoking-related and income-related inequalities in both self-reported and lifestyle-related ill-health. The results suggest that smoking and alcohol use contribute positively to income-related inequality in health. Smoking participation accounts for up to 7.35% of all measured inequality in health and 3.11% of the inequality in self-reported health. The estimates are generally higher for all measured inequality in health (up to 14.67%) when smoking duration is considered. Alcohol consumption accounts for 27.83% of all measured inequality in health and 3.63% of the inequality in self-reported health.

**Conclusion:**

This study provides evidence that inequalities in both self-reported and lifestyle-related ill-health are highly prevalent within smokers and the poor. These inequalities need to be explicitly addressed in future programme planning to reduce health inequalities in South Africa. We suggest that policies that can influence poor individuals to reduce tobacco consumption and harmful alcohol use will improve their health and reduce health inequalities.

## Background

Many low- and middle-income countries are experiencing an epidemiological transition from communicable to non-communicable diseases [7]. This has negative consequences for their human capital development, and imposes a growing economic burden on their societies [26]. While the prevalence of such diseases varies with socioeconomic status, the inequalities can be exacerbated by adopted lifestyles of individuals. Evidence based assessment of this relationship is useful for policies towards reducing unhealthy behaviours. A number of studies have examined the effects and contributions of lifestyle factors such as tobacco use, harmful use of alcohol and obesity on income-related inequalities in health [4, 45]. Evidence from these studies has been important for the formulation of anti-smoking and alcohol policies. While such research provides evidence on the overall contribution of these factors on income-related inequality in self-reported health (SRH) and mortality, they do not explore their contribution to specific lifestyle-related diseases. In addition, self-reported health is a subjective evaluation of people’s general health status [34] rather than an objective measure [57]. This paper expands the analysis of the contributions of smoking and alcohol consumption to income-related inequality in health by incorporating more objective measures of health that are directly associated to these lifestyle practices. The main argument in this paper is that using SRH as a measure underestimates the contributions of smoking and alcohol use to income-related inequality in health. It should be noted that self-reported health and self-assessed health are used interchangeably in this paper.

The idea from the growing body of literature is that the gradient in inequality in health between the poor and the rich is likely to depend on differences in lifestyle. The hypothesis is that unhealthy practices have negative health effects and if concentrated among the poor, socioeconomic-related inequalities in health will widen [10, 13, 45]. In this regard, the important contribution of education, occupation, and age [27] and social determinants of health [2] on income-related inequality in health have been examined empirically. Studies that have attempted to examine the contribution of lifestyle factors such as smoking, alcohol use and obesity on income-related health inequality, have done so without considering health outcomes that may be directly associated with these factors [38, 45]. This paper contributes to the existing literature by considering health outcomes that are directly associated with smoking and harmful alcohol use.

Globally, over 63% of all deaths are attributable to non-communicable diseases (NCDs), and over 6 million premature deaths each year are attributed to smoking-related ill-health, making tobacco use the leading avoidable risk factor for NCDs [18, 33, 54]. While reducing premature mortality from NCDs is now on the post-2015 development agenda, it is estimated that by 2030, deaths from NCDs will be five times higher than deaths from communicable diseases in low- and middle-income countries [36]. The rapid acceleration of the NCDs is mainly due to lifestyle changes, including smoking and harmful alcohol use. In South Africa, it is estimated that tackling lifestyle risk factors associated with NCDs could reduce premature disability and mortality by 20% [3]. The prevention of NCDs is considerably more effective and less costly than their treatment [12]. The priority of many health care systems, including the National Health System in South Africa, is to mitigate inequalities in health, partly by reducing unhealthy behaviours of individuals. Smoking and alcohol interventions since the democratic transition in 1994, in South Africa have reduced levels of smoking and harmful alcohol use, but the declines are not evenly distributed across income quintiles (see Table 1). This necessitate the need to examine how the uneven distribution of the decline in smoking and alcohol use contribute to inequality in related health outcomes.

**Table 1:** The distribution of disease burden by income quintile

	Wave 1							Wave 2						
Diease Type	Poorest	Poor	Middle	Rich	Richest	Unweighted	Weighted	Poorest	Poor	Middle	Rich	Richest	Unweighted	Weighted
Independent Variables (smoking and Alcohol use)														
Individual is a current smoker	16.59	21.53	25.04	23.21	13.63	3,279	5,967,853	18.55	24.47	25.79	21.00	10.18	2,652	5,390,039
Individual drinks alcohol	15.29	19.56	23.48	22.19	19.47	5,362	10,409,442	19.13	22.90	24.41	20.34	13.22	4,773	10,134,185
Dependent Variables (Health outcomes)														
Diagnosed of tuberculosis	25.86	26.72	26.29	17.10	04.02	696	1,017,966	25.00	31.68	21.89	17.86	03.57	644	978,551
Have high blood pressure	17.20	23.66	2.27	20.81	13.06	2,489	3,819,946	17.52	25.46	25.22	20.97	10.84	2,141	3,541,095
Diagnosed of diabetes	12.19	21.58	26.19	25.04	14.99	607	987,401	16.79	25.34	23.97	20.92	12.98	655	1,108,548
Diagnosed of stroke	16.03	25.64	28.85	19.23	10.26	156	251,013	16.67	27.08	25.00	23.61	07.64	144	215,720
Diagnosed of heart diseases	15.51	23.06	23.47	22.45	15.51	490	837,918	15.64	26.07	23.31	21.78	13.19	326	573,867
Diagnosed of cancer	09.78	14.13	16.30	20.65	39.13	92	201,808	14.67	18.67	12.00	20.00	34.67	75	214,723
Have persistent cough	22.43	25.57	24.11	18.97	08.92	1,850	3,093,475	20.98	25.55	24.36	20.98	08.12	1,687	3,317,332
Experienced depression	27.22	25.53	22.24	16.75	08.26	7,387	12,934,471	28.45	27.57	21.60	15.83	06.55	6,583	11,200,000
Experienced chest pain	24.70	28.23	24.42	17.47	05.18	1,757	2,786,539	23.34	29.43	24.05	17.88	05.30	1,264	2,095,576
Self-reported poor health	22.37	26.64	25.89	19.38	05.72	3,303	4,975,161	23.02	28.80	24.73	17.91	05.54	2,111	3,174,550
	Wave 3							Wave 4						
	Poorest	Poor	Middle	Rich	Richest	Unweighted	Weighted	Poorest	Poor	Middle	Rich	Richest	Unweighted	Weighted
Independent Variables (smoking and Alcohol use)														
Individual is a current smoker	17.99	25.42	26.07	19.62	10.89	3,241	6,209,227	19.38	25.19	26.69	19.33	9.40	4,267	7,115,620
Individual drinks alcohol	18.72	24.72	23.19	19.08	14.30	5,887	11,350,752	20.40	24.75	23.79	19.10	11.96	9,240	16,064,962
Dependent Variables (Health outcomes)														
Diagnosed of tuberculosis	27.06	29.88	22.94	15.69	04.43	994	1,499,848	27.20	29.33	23.63	14.01	05.82	842	1,190,834
Have high blood pressure	20.04	26.52	24.22	18.02	11.20	3,224	5,096,330	23.14	25.87	23.42	17.64	09.93	2,165	3,373,811
Diagnosed of diabetes	19.65	25.47	21.74	19.42	13.72	860	1,449,373	17.97	24.96	20.63	21.30	15.14	601	1,239,479
Diagnosed of stroke	17.07	31.22	26.34	19.51	05.85	205	265,006	26.36	28.64	27.73	12.73	04.55	220	327,007
Diagnosed of heart diseases	17.56	27.54	21.56	21.96	11.38	501	816,789	23.59	25.90	25.90	15.64	08.97	390	634,708
Diagnosed of cancer	10.68	28.16	17.48	25.24	18.45	103	192,772	23.19	25.60	17.87	16.43	16.91	207	459,566
Have persistent cough	24.04	29.88	23.70	15.03	08.16	3,274	5,030,600	27.42	26.46	23.26	15.75	07.11	2,812	4,436,501
Experienced depression	28.61	28.01	21.10	15.50	06.78	8,272	13,100,000	26.66	25.71	21.95	17.03	08.65	10,103	15,600,000
Experienced chest pain	26.57	29.41	22.48	15.55	06.00	1,833	2,722,351	26.58	27.06	24.17	15.74	06.45	2,077	3,271,733
Self-reported poor health	22.64	29.63	26.07	15.47	06.20	2,275	3,481,263	25.35	28.67	24.36	14.60	09.45	2,275	3,797,960

Evidence still points to wide inequalities in the distribution of health in South Africa, with those at the top end of the socioeconomic scale having better health outcomes [1, 2]. Two in every five deaths in South Africa are related to NCDs, with a high prevalence attributed to avoidable risk factors such as tobacco use and alcohol consumption [52]. Socioeconomic-related inequalities in health are particularly widened by the ongoing prevalence of NCDs among poor South Africans, and the likely consequence of health-damaging behaviours and living conditions. While there has been a decline in both smoking and per capita alcohol consumption in the last two decades, there is little evidence on how this has affected inequalities in health, and whether income-related health inequality from such behaviours are concentrated among the poor or the rich. An analysis of the contributions of tobacco and alcohol use to health is essential for policies that can improve health outcomes and reduce health inequalities and the growing economic burden of risky lifestyles in developing countries.

The optimal level of alcohol consumption is not zero, since it has both beneficial and harmful effects on health. Evidence suggests that overall, harmful and excessive alcohol consumption is the third most important risk factor contributing to NCDs, injuries, and communicable diseases [53]. The effects of alcohol use on health are dependent on the pattern of drinking and the volume of alcohol consumed. In South Africa, alcohol consumption has a long social history and the industry is now an integral part of the economy, creating employment opportunities and contributing about 1.7% to government revenue each year. The costs of drunken driving accidents, alcohol-related medical costs, alcohol-induced domestic violence, and premature death from alcohol induced illnesses have made the industry responsible for much misery in the country. The adult per capita consumption is 11 l of pure alcohol and the average consumption per drinker of about 27.1 l of absolute alcohol is among the highest in the world [53]. Over 45% of drinkers in South Africa are weekly heavy episodic drinkers, showing a peculiar and hazardous pattern of drinking [51].

## Data and Method

The analysis is based on data from the four waves of the National Income Dynamic Study (NIDS). This includes Wave 1 (conducted in 2008), Wave 2 (2010–2011), Wave 3 (2012) and Wave 4 (2014–2015). NIDS is a national representative panel survey repeated with the same household members every two years, tracking changes in the well-being of individuals and households over time. Individuals are interviewed on a range of topics, including their socioeconomic status, disease profile, and lifestyle attributes. Our analysis focuses on individuals aged 15 years and older. The sample sizes vary across waves, depending on the number of Continuing Sample Members and Temporary Sample Members. We consider the four available waves to explore the dynamics between income-related health inequality and cigarette smoking and alcohol consumption.

### Measurement of income

We use two measures of income, namely, household per capita income, and household income per equivalent adult. Using aggregate income of households to make comparisons may be deceptive, since households differ in size and demographic composition. Comparison of household income requires some form of normalisation. The simplest way is by comparing household per capita income by taking household total income as a ratio of household size. The assumption in this approach is that the cost of a child is equivalent to the cost of an adult. However, a more complex approach is required to control for household demographic make-up. We do this by converting household income to household income per “equivalent adult”. This method assumes that the general cost of a child is smaller than the cost of an additional adult.

For household income per adult equivalent, if *E* is an index for household needs, then *E* is likely to depend on age of household members and the household size. If *AE* is the adult equivalent household income and *X* is the unadjusted household income, then *AE* = ^*X*^ */E*. While there are several formulations for *E*, we use the double parameter class of equivalence scales of Cutler and Katz [14], given by *E* = (*N*_*A*_ + *cN*_*c*_)^*θ*^. Where *N*_*A*_ is for adults and *N*_*c*_ is for children; *c* is a parameter for the cost of a child relative to that of an adult and *θ* measures overall economies of scales within the household. Children are counted as adults if *c* = 1 [11]. In most cases, the values of *c* and *θ* are between 0 and 1. In the context of South Africa, most researchers tend to set *c* = 0*.*5 and *θ* = 0*.*9 as proposed by Deaton in 1993 (see, [37]). Using a variety of combinations of *c* and *θ* for meaningful comparisons by Woolard and Leibbrandt [56] the results were not significantly different from the bench marked values of *c* = 0*.*5 and *θ* = 0*.*9.

### Measurement of health

We explore the contribution of cigarette smoking and alcohol consumption to income-related health inequality using a range of health indicators. The indicators are selected based on their availability in the data-sets used and their likely association with the chosen health-related behaviours (smoking and harmful alcohol use). The following health outcomes were analysed: Diagnosed with tuberculosis, diagnosed with high blood pressure, diagnosed with diabetes, diagnosed with stroke, diagnosed with heart problems, diagnosed with cancer, having persistent cough, experiencing depression, and experiencing chest pain. Information on these indicators, though reported by the respondents, are based on medical diagnoses, and can be regarded as objective measures of health. All health measures are defined as binary outcomes equivalent to 1 if the respondent reported to be diagnosed of a particular disease. For a more generic measure, we use principal component analysis to reduce these indicators to a single index value for health status.

The World Health Organisation (WHO) defines health as the state of complete physical, mental, and social well-being, and not just the absence of disease or infirmity. Based on this definition, it is difficult to find a measure that collapses the separate dimensions of health into one construct. The international literature indicates that individuals consider all dimension of health when asked to evaluate their health. The literature shows that self-assessed health is a strong predictor of mortality and health care utilisation [6, 28, 30, 46]. Self-assessed health is one of the closest measures that captures all dimensions of health, and is frequently used in health inequality literature. We conduct a sensitivity analysis by including self-assessed health as part of our health measure. This variable takes the value 1 if an individual reported excellent, very good or good health and zero if they report their health as fair or poor. The results are consistent across all measures, with individuals from poor households more likely to report ill health than those from rich households, and with alcohol and tobacco consumption contributing positively to income-related inequality in health.

### Alcohol- and smoking-related disease profiles

In this section we discuss how the considered health conditions are associated with smoking and alcohol use. The bioactive compounds in nicotine have far-ranging effects on human health. For example, nicotinic receptors are found not only in the brain, but throughout the body, such as in muscle, lungs, kidneys, and skin [29, 31]. Evidence suggests a strong and positive relationship between smoking and alcohol use on tuberculosis [23, 32], with greater risk among individuals who are both smokers and drinkers [21]. Murray et al. [39] showed that smoking is a strong predictor of lung cancer, and the associated risk from smokeless tobacco is less than the risk from smoking traditional tobacco products [9]. The risk of having cardiovascular heart diseases is high at all levels of cigarette smoking, even at fewer than five cigarettes per day [42]. Evidence from epidemiologic and pathogenesis studies support a potential causal relationship between smoking and type 2 diabetes [58]. Even after controlling for age, hypertension, and cardiovascular disease risk factors, Wolf et al. [55] showed that smoking was significantly related to the incidence of stroke. Using a structural equation modelling, Boden et al. [8] suggest that nicotine dependence leads to increased risk of depression.

Unlike smoking, alcohol use has both beneficial and detrimental effects on diabetes and some cardiovascular diseases, depending on the patterns and volumes of alcohol consumed. Evidence suggests a strong relationship between ethanol and cancers [41]. Systematic reviews have shown that alcohol consumption increases the risk of developing cancer (see [19, 43]). The effects of alcohol use on diabetes are dose dependent and the risk of type 2 diabetes reduces with moderate alcohol use (see [5]). Excessive alcohol consumption may increase body weight, the concentration of fats in the blood, and blood pressure [50]. A review by Rehm et al. [40] shows the risk of depression is two- to three-fold higher among alcohol users. Harmful alcohol use affects multiple aspects of the cardiovascular system, and increases the risk of hypertension, heart disease, and stroke [22].

### Measure of health inequality

We use the health concentration index (CI) to examine the extent of income-related inequality in the distribution of ill-health across the population [48]. Unlike the Gini index, which measures inequalities in health, the CI is a bivariate measure of inequality in health status related to the ranking of (an)other variable(s) (in our case income or smoking). The CI lies between −1 and +1, and it takes a positive value when income-related inequality favours the rich and negative values if it favours the poor [48]. The value of CI will be zero, if the population’s ill-health is evenly concentrated along the distribution of income or if, on average, the positive and negative effects across the distribution cancel out. The index is −1 if all ill-health in a population is concentrated among the poor and +1 if all ill-health is concentrated among the rich (see, [1, 44]).

The advantage of the standard CI is that it provides the possibility of summarising the extent of inequality in a single measure that can be used to compare inequality levels over time, and across countries and groups. However, the standard CI may not be a good measure for comparing inequality between countries and over time, if the health indicators are bounded [47]. For dichotomous outcome variables, the bounds of the CI depend on the mean (*μ*) of the variable and lies between *μ* − 1 and 1 − *μ*. While Wagstaff [47], suggests normalisation of the CI using (1 − *μ*), Erreygers [16] argues that this is an *ad hoc* procedure and proposes the use of a corrected concentration index (CCI) with the claim that it satisfies level independence (that is an equal increment of health for all individuals does not affect the value of the index). All health outcomes used in this paper are binary and bounded in nature (between 0 and 1), and therefore to be able to compare our inequality indices over time, we use the CCI proposed by [16]. The CCI is written as:

1 $$ C C I=\frac{4\mu}{b- a}* C $$

Where *μ* is mean health status, *C* is the standard CI, *b* is the maximum level of health (1) and *a* is the minimum level of the health variable (0).

### Decomposition of the CCI by Factors (alcohol use and smoking)

The standard CI can be decomposed to net out the contribution of different covariates to income-related health inequality using a regression technique [15, 49]. The CCI is a modification of the standard CI, to satisfy the desired properties of a rank dependent index, and can be decomposed using the same technique as the CI. First, the regression analysis expresses the health variables as a function of its determinants as follows:

2 $$ {h}_i=\alpha +{\sum}_k{\beta}_{k i}{x}_{k i}+{\varepsilon}_i $$

Where *h*_*i*_ is the health status of individual *i*, *x*_*k*_ is a set of demographic characteristics, and socioeconomic factors, including cigarette smoking and alcohol consumption, *α* is the constant and *ε*_*i*_ is the error term. The decomposed CCI is the weighted sum of the CI for each health covariate. The weights are the partial effects and the CCI can be re-written as:

3 $$ C C I=4*\left[{\displaystyle \sum \left({\beta}_k G{C}_k\right)+ G{C}_{\varepsilon}}\right]=4*\left[{\displaystyle \sum_k\left({\beta}_k{\overline{x}}_k C{I}_k\right)+ G{C}_{\varepsilon}}\right] $$

Where ¯*x*_*k*_ and *CI*_*k*_ are the means of *x*_*k*_ and CI respectively, *GC*_*k*_ and *GC*_*ε*_ are the generalised concentration indices for *x*_*k*_ and the error term. This allows us to estimate the contribution of cigarette smoking and alcohol consumption to income-related inequalities in health. The overall contribution of each of these factors to income-related inequalities in the respective health outcomes is the product of three separate components, namely, the coefficient (*β*_*k*_); the prevalence of each variable given by its mean (*x* ^¯^_*k*_); and the distribution of the variable across income groups, given by the concentration index (*CI*_*k*_)*,* multiplied by four.

The approaches for decomposing bivariate rank dependent indices [16, 49] are one-dimensional, ignoring the covariance between health and income [17]. In addition, most of these approaches focus on procedures that give little thought to identification strategies [20], are not explicit about the parameter of interest, and require identifying assumptions [17, 24, 35]. To address these limitations, Erreygers and Kessels [17] and Kessels et al. [35] develop a set of two-dimensional indices that consider the covariance between health and income. Heckley et al. [24] also propose the use of a regression-based decomposition of a bivariate rank dependent index that relaxes some identifying assumptions of the decomposition of Wagstaff et al. [49], stating clearly the parameter of interest and the underlying assumptions. This approach is a suitable method for determining the causal effect of a covariate on the index, and a useful descriptive decomposition method when no causal inference is made, but relies on a suitable identification strategy [24]. These approaches are potential ways to acknowledge the bivariate nature of such inequality indices. However, the requirements of such structural equation modelling are data demanding (it requires two instrumental variables for health and for rank (income) respectively) limiting their application (see [24]).

## Results

The means and smoking-related health inequality indices of each health indicator by wave, presented in Table 2 provide interesting basis for over time comparison. The means of some health indicators, such as tuberculosis, stroke, and cancer, are higher for respondents in Wave 4 than in Wave 1, but are lower for high blood pressure, heart diseases, and self-reported health. Depression has the highest mean values, ranging from 40% in Wave 2 to 51% in Wave 4, and cancer has the lowest mean values, ranging from 0.3% in Wave 2 to 2% in Wave 4. The proportion of those with self-reported poor health decreased by 28% between Wave 1 and Wave 4, indicating an improvement in health over the period. Conversely, the health index for smoking-related diseases suggests a deterioration over the period. The results show that there is a smoking gradient in health, for all four waves, for both smoking intensity (average number of cigarettes smoked a day) and smoking duration (the number of years an individual has been a smoker). NIDs did not collect data on duration and intensity of alcohol consumption. This limits the assessment of alcohol-related inequality in health.

**Table 2:** Mean health and smoking-related health inequality by disease type and by wave

Panel A	Wave 1						Wave 2					
	All smokers		Smoking intensity		Smoking duration		All smokers		Smoking intensity		Smoking duration	
Variable	Obs	Mean	CCII	SE	CCIage	SE	Obs	Mean	CCII	SE	CCIage	SE
Diagnosed of tuberculosis	3,271	0.048	0.001	(0.009)	0.060***	(0.009)	2,640	0.044	0.038***	(0.009)	0.037***	(0.010)
Have high blood pressure	3,274	0.112	0.053***	(0.013)	0.233***	(0.012)	2,636	0.077	0.043***	(0.013)	0.195***	(0.013)
Diagnosed of diabetes	3,267	0.021	0.021***	(0.006)	0.057***	(0.007)	2,633	0.019	0.018**	(0.007)	0.053***	(0.007)
Diagnosed of stroke	3,268	0.008	0.000	(0.004)	0.016***	(0.004)	2,645	0.007	0.001	(0.004)	0.015***	(0.003)
Diagnosed of heart diseases	3,266	0.026	0.028***	(0.006)	0.054***	(0.006)	2,638	0.015	0.009	(0.006)	0.038***	(0.006)
Diagnosed of cancer	3,265	0.005	0.019***	(0.004)	0.022***	(0.004)	2,643	0.003	0.002	(0.004)	0.021***	(0.004)
Have persistent cough	3,259	0.131	0.028**	(0.013)	0.077***	(0.013)	2,646	0.166	−0.005	(0.016)	0.095***	(0.017)
Experienced depression	3,270	0.460	−0.065***	(0.019)	−0.021	(0.019)	2,632	0.395	−0.168***	(0.21)	−0.065***	(0.022)
Experienced chest pain	3,253	0.114	0.008	(0.012)	0.082***	(0.013)	2,648	0.104	−0.013	(0.013)	0.099***	(0.014)
Self-reported poor health	3,259	0.176	0.024***	(0.015)	0.270***	(0.015)	2,651	0.101	−0.001	(0.014)	0.145***	(0.014)
Health index	3,207	−0.078	0.019***	(0.006)	0.089***	(0.005)	2,562	0.086	−0.002	(0.007)	0.085***	(0.007)
Panel B	Wave 3						Wave 4					
	All smokers		Smoking intensity		Smoking duration		All smokers		Smoking intensity		Smoking duration	
	Obs	Mean	CCII	SE	CCIage	SE	Obs	Mean	CCI	SE	CCIage	SE
Diagnosed of tuberculosis	3,235	0.072	0.010	(0.010)	0.056***	(0.010)	4,038	0.056	0.021***	(0.008)	0.022***	(0.008)
Have high blood pressure	3,232	0.139	0.067***	(0.014)	0.280***	(0.013)	3,897	0.098	0.025**	(0.011)	0.186***	(0.011)
Diagnosed of diabetes	3,231	0.030	0.048***	(0.008)	0.093***	(0.008)	4,176	0.027	0.022***	(0.006)	0.073***	(0.006)
Diagnosed of stroke	3,235	0.006	0.004	(0.003)	0.012***	(0.003)	4,240	0.010	0.003	(0.004)	0.032***	(0.004)
Diagnosed of heart diseases	3,238	0.041	0.026***	(0.008)	0.086***	(0.008)	4,179	0.020	0.014***	(0.005)	0.039***	(0.005)
Diagnosed of cancer	3,236	0.007	0.012**	(0.003)	0.012***	(0.003)	4,250	0.016	0.035***	(0.005)	0.010**	(0.004)
Have persistent cough	3,239	0.211	0.023	(0.016)	0.118***	(0.016)	4,265	0.168	0.015	(0.012)	0.059***	(0.012)
Experienced depression	3,222	0.451	−0.064***	(0.019)	−0.022	(0.020)	4,263	0.505	−0.071***	(0.017)	−0.022	(0.016)
Experienced chest pain	3,239	0.112	0.000	(0.012)	0.042***	(0.013)	4,264	0.128	0.012	(0.011)	0.093***	(0.011)
Self-reported poor health	3,238	0.137	0.061***	(0.013)	0.203***	(0.013)	4,262	0.126	0.022**	(0.011)	0.193***	(0.011)
Health index	3,185	0.117	0.023***	(0.006)	0.087***	(0.006)	3,606	0.185	0.006**	(0.017)	0.050***	(0.003)

Notes: Results presented in this table are corrected concentration indices for smoking-related health inequality. The health indicators are all binary outcomes equivalent to 1 if the respondent is diagnosed of a given disease. The health index is continuous with high values representing poor health outcomes. The cigarette smoking variables are both continuous. Positive values of smoking-related health inequality indices indicate that poor health is concentrated among heavy smokers and those with longer smoking duration. ***Statistically significant at the 1% level; statistically significant at the 5% level; *statistically significant at the 10% level

The corrected concentration indices for both smoking intensity and smoking duration are generally positive and significantly different from zero, indicating that poor health is concentrated among heavy smokers and those with longer smoking duration. There are substantial differences in the level of inequality between health indicators and across waves. The magnitudes of the inequality estimates are generally higher when smoking duration rather than smoking intensity is used and are consistent between the health indicators and across waves. Smoking-related inequality in self-reported health decreased from 0.270 in Wave 1 to 0.193 in Wave 4 for smoking duration and from 0.024 to 0.022 for smoking intensity. Similarly, smoking-related inequality in the health index decreased from 0.089 in Wave 1 to 0.050 in Wave 4 for smoking duration and from 0.019 to 0.006 for smoking intensity. This confirms that tobacco consumption is hazardous at all levels and smoking-related health effects are more time than intensity dependent.

The means and income-related health inequality estimates for each health indicator by wave are reported in Table 3. The proportion of respondents self-reporting poor health decreased from 0.177 in Wave 1 to 0.108 in Wave 4, indicating an improvement in average health status. Using the health index, we find that the means range between −0.125 and 0.034, indicating an increase prevalence of non-communicable diseases (note that higher values of the index signify poor health). The distribution of smoking prevalence, alcohol consumption, and disease burden by income quintile is presented in Table 1. The prevalence of smoking and alcohol use is primarily among those in the lower income quintiles. On the other hand, those in the highest income quintile (richest) are less than proportionally represented in the prevalence of smoking and alcohol use.

**Table 3:** Mean health and income-related health inequality by disease type and by wave

Panel A: Using household per capita income																
	Wave 1				Wave 2				Wave 3				Wave 4			
Variables	Obs	Mean	CCI	SE	Obs	Mean	CCI	SE	Obs	Mean	CCI	SE	Obs	Mean	CCI	SE
Diagnosed of tuberculosis	15,552	0.036	−0.028***	(0.003)	17,551	0.031	−0.020***	(0.003)	18,673	0.047	−0.028***	(0.004)	21,937	0.035	−0.025***	(0.003)
Have high blood pressure	15,562	0.135	0.023***	(0.006)	17,522	0.113	0.038***	(0.006)	18,666	0.161	0.043***	(0.006)	20,358	0.106	0.023***	(0.005)
Diagnosed of diabetes	15,527	0.035	0.024***	(0.003)	17,536	0.035	0.021***	(0.003)	18,638	0.046	0.028***	(0.004)	22,034	0.036	0.034***	(0.003)
Diagnosed of stroke	15,527	0.009	−0.004**	(0.002)	17,564	0.007	0.002	(0.001)	18,682	0.008	−0.004**	(0.002)	22,575	0.009	−0.002	(0.001)
Diagnosed of heart diseases	15,514	0.030	−0.013***	(0.003)	17,536	0.018	−0.012***	(0.002)	18,673	0.026	−0.014***	(0.003)	22,356	0.018	−0.002	(0.002)
Diagnosed of cancer	15,505	0.007	0.013***	(0.002)	17,552	0.007	0.010***	(0.001)	18,686	0.006	0.005***	(0.001)	22,648	0.013	0.014***	(0.002)
Have persistent cough	15,535	0.110	−0.009	(0.006)	16,848	0.112	−0.023***	(0.006)	18,695	0.159	−0.016**	(0.006)	22,737	0.127	−0.025***	(0.005)
Experienced depression	15,536	0.459	−0.184***	(0.009)	16,720	0.381	−0.086***	(0.007)	18,630	0.414	−0.161***	(0.008)	22,742	0.447	−0.083***	(0.008)
Experienced chest pain	15,525	0.099	−0.047***	(0.006)	16,847	0.070	−0.018***	(0.005)	18,696	0.086	−0.031***	(0.005)	22,735	0.093	−0.030***	(0.004)
Self-reported poor health	15,535	0.177	−0.074***	(0.007)	17,600	0.101	−0.026***	(0.005)	18,698	0.110	−0.023***	(0.005)	22,744	0.108	−0.018***	(0.004)
Health index	15,296	−0.125	−0.019***	(0.003)	16,342	−0.050	−0.001	(0.002)	18,415	−0.089	−0.007***	(0.002)	19,220	0.034	−0.003**	(0.001)
Panel B: Using household per capita income by adult equivalent																
	Wave 1				Wave 2				Wave 3				Wave 4			
Variables	Obs	Mean	CCI	SE	Obs	Mean	CCI	SE	Obs	Mean	CCI	SE	Obs	Mean	CCI	SE
Diagnosed of tuberculosis	15,552	0.036	−0.032***	(0.003)	17,551	0.031	−0.023***	(0.003)	18,673	0.047	−0.031***	(0.004)	21,937	0.035	−0.034***	(0.003)
Have high blood pressure	15,562	0.135	0.025***	(0.006)	17,522	0.113	0.038***	(0.006)	18,666	0.161	0.038***	(0.006)	20,358	0.106	0.022***	(0.005)
Diagnosed of diabetes	15,527	0.035	0.025***	(0.003)	17,536	0.035	0.021***	(0.003)	18,638	0.046	0.027***	(0.004)	22,034	0.036	0.033***	(0.003)
Diagnosed of stroke	15,527	0.009	−0.006***	(0.002)	17,564	0.007	0.001	(0.001)	18,682	0.008	−0.005***	(0.002)	22,575	0.009	−0.002	(0.001)
Diagnosed of heart diseases	15,514	0.030	−0.011***	(0.003)	17,536	0.018	−0.011***	(0.002)	18,673	0.026	−0.010***	(0.002)	22,356	0.018	−0.000	(0.002)
Diagnosed of cancer	15,505	0.007	0.013***	(0.002)	17,552	0.007	0.010***	(0.001)	18,686	0.006	0.005***	(0.001)	22,648	0.013	0.013***	(0.002)
Have persistent cough	15,535	0.110	−0.017***	(0.006)	16,848	0.112	−0.022***	(0.006)	18,695	0.159	−0.026**	(0.006)	22,737	0.127	−0.029***	(0.005)
Experienced depression	15,536	0.459	−0.187***	(0.009)	16,720	0.381	−0.094***	(0.009)	18,630	0.414	−0.167***	(0.008)	22,742	0.447	−0.085***	(0.008)
Experienced chest pain	15,525	0.099	−0.047***	(0.006)	16,847	0.070	−0.020***	(0.005)	18,696	0.086	−0.035***	(0.005)	22,735	0.093	−0.040***	(0.004)
Self-reported poor health	15,535	0.177	−0.079***	(0.007)	17,600	0.101	−0.030***	(0.005)	18,698	0.110	−0.029***	(0.005)	22,744	0.108	−0.025***	(0.005)
Health index	15,296	−0.125	−0.021***	(0.003)	16,342	−0.050	−0.004	(0.002)	18,415	−0.089	−0.010***	(0.002)	19,220	0.034	−0.004***	(0.001)

Notes: Results presented in this table are corrected concentration indices for income-related health inequality. The health indicators are all binary outcomes equivalent to 1 if the respondent is diagnosed of a given disease. The health index is continuous with high values representing poor health outcomes. The income variables are both continuous. Negative values of the concentration indices indicate that poor health is concentrated among individuals from low-income households. ***Statistically significant at the 1% level; statistically significant at the 5% level; *statistically significant at the 10% level. Obs is observations and SE are standard error of the CCI

It is evident from Table 1 that the prevalence of lifestyle-related diseases is higher among individuals in the lower income quintiles. This is seen over time with the exception of cancer which is more prevalent among those in the highest income quintile. The concentration indices for most of the health indicators in Table 3 are negative, indicating a concentration of ill-health among the poor. For example, the concentration indices for the health index range from −0.019 in Wave 1 to −0.003 in Wave 4, indicating a decline in income-related inequality over time. Generally, the inequality indices are higher when household per capita income is used than when the per capita income by adult equivalent is used. However, the results are consistent across the two measures, indicating that there is an income gradient in health that varies among diseases and across time.

In Table 4, we summarise the percentage contributions of cigarette and harmful alcohol use to observed income-related health inequality. The contribution of each variable can be positive or negative, depending on the sign of its health effects and its distribution by income (shown by the sign of the CCI). A positive (negative) percentage contribution of each covariate implies that *ceteris paribus*, income-related health inequality will be lower if the covariate is equally distributed across income groups, or the covariate has a zero-health elasticity. Smoking accounts for 3.02 to 7.35% of all measured inequality in the health index in Wave 1 and Wave 4, respectively, and a maximum of 3.11% for self-reported health (see Wave 3 data in Table 4). Alcohol use accounts for 15.44 to 27.83% of all measured inequality in the health index in Wave 1 and Wave 4, respectively, with a maximum of 3.63% for self-reported health (see Wave 2 of Table 4). Using the EQ-5D^1^ as a measure of health, Vallejo-Torres and Morris [45] obtains a maximum contribution of 2.3% for smoking in for UK. While the percentage contributions of smoking and alcohol use to individual diseases are small, their overall effect on health (measured by the health index) is larger.

**Table 4:** The contribution of Smoking and alcohol to income-related inequalities by disease type and by wave

	Wave 1						Wave 2					
	Cigarette smoking			Alcohol consumption			Cigarette smoking			Alcohol consumption		
	Elasticity	Contribution	%	Elasticity	Contribution	%	Elasticity	Contribution	%	Elasticity	Contribution	%
Diagnosed of tuberculosis	−0.0005	−0.0002	−0.1981	0.0080	0.0085	3.1960	0.002	0.001	0.769	0.004	0.005	1.738
Have high blood pressure	−0.0053	−0.0019	−2.1114	−0.0007	−0.0007	−0.2766	−0.005	−0.002	−2.099	−0.002	−0.002	−0.854
Diagnosed of diabetes	−0.0028	−0.0010	−1.1269	−0.0025	−0.0026	−0.9862	−0.003	−0.001	−1.152	−0.003	−0.003	−1.146
Diagnosed of stroke	−0.0011	−0.0004	−0.4474	0.0007	0.0008	0.2856	0.001	0.000	0.248	−0.002	−0.002	−0.741
Diagnosed of heart diseases	−0.0013	−0.0005	−0.5236	−0.0011	−0.0011	−0.4260	0.000	0.000	0.057	−0.001	−0.001	−0.323
Diagnosed of cancer	−0.0011	−0.0004	−0.4506	0.0009	0.0009	0.3508	−0.001	−0.000	−0.271	−0.001	−0.001	−0.460
Have persistent cough	0.0034	0.0012	1.3407	0.0095	0.0102	3.8118	0.007	0.002	2.646	0.016	0.017	6.392
Experienced depression	0.0044	0.0016	1.7559	0.0085	0.0091	3.4084	0.004	0.001	1.722	0.018	0.020	7.339
Experienced chest pain	0.0039	0.0014	1.5659	0.0071	0.0076	2.8520	0.006	0.002	2.394	0.010	0.010	3.806
Self-reported poor health	−0.0007	−0.0002	−0.2776	0.0091	0.0097	3.6295	0.002	0.001	0.607	0.003	0.004	1.389
Health index	0.0075	0.0027	3.0176	0.0386	0.0412	15.4396	0.0206	0.0070	8.1585	0.0487	0.0518	19.4776
	Wave 3						Wave 3					
	Cigarette smoking			Alcohol consumption			Cigarette smoking			Alcohol consumption		
	Elasticity	Contribution	%	Elasticity	Contribution	%	Elasticity	Contribution	%	Elasticity	Contribution	%
Diagnosed of tuberculosis	0.0029	0.0009	1.1496	0.0045	0.0035	1.7942	0.0005	0.0001	0.2067	0.0087	0.0067	3.4760
Have high blood pressure	−0.0028	−0.0008	−1.1220	−0.0039	−0.0030	−1.5486	−0.0032	−0.0007	−1.2624	0.0078	0.0060	3.1219
Diagnosed of diabetes	−0.0035	−0.0010	−1.3990	−0.0055	−0.0043	−2.2132	−0.0059	−0.0013	−2.3602	0.0056	0.0043	2.2349
Diagnosed of stroke	−0.0008	−0.0002	−0.3129	0.0008	0.0006	0.3026	−0.0001	−0.0000	−0.0388	0.0003	0.0002	0.1137
Diagnosed of heart diseases	0.0046	0.0014	1.8397	−0.0023	−0.0018	−0.9134	0.0024	0.0005	0.9456	0.0016	0.0012	0.6467
Diagnosed of cancer	0.0002	0.0001	0.0898	0.0001	0.0001	0.0326	0.0025	0.0005	0.9980	0.0002	0.0001	0.0750
Have persistent cough	0.0136	0.0041	5.4591	0.0083	0.0065	3.3263	0.0084	0.0018	3.3559	0.0109	0.0083	4.3491
Experienced depression	0.0085	0.0025	3.3891	0.0170	0.0133	6.7845	0.0132	0.0028	5.2920	0.0115	0.0088	4.6195
Experienced chest pain	0.0069	0.0021	2.7404	0.0032	0.0025	1.2825	0.0053	0.0011	2.1375	0.0108	0.0083	4.3228
Self-reported poor health	0.0078	0.0023	3.1100	0.0006	0.0005	0.2311	0.0055	0.0012	2.1941	0.0034	0.0026	1.3667
Health index	0.0168	0.0132	6.7222	0.0532	0.0160	21.2877	0.0184	0.0039	7.3467	0.0696	0.0533	27.8292

Notes: Results presented in this table are elasticities, contributions, and percentage contributions of cigarette smoking and alcohol consumption to income-related health inequality. The results are obtained by decomposing the income-related health inequality indices into health related covariates, including smoking and alcohol use. The health indicators are all binary outcomes equivalent to 1 if the respondent is diagnosed of a given disease. The health index is continuous with high values representing poor health outcomes. The tobacco and alcohol use variables are both binary equal to 1 if the respondent is a current smoker or drink regularly. Other covariates include household per capita income, gender, categories for age, province of residence, race, marital status, and education

Our data indicates that some individuals are both smokers and drinkers, and others are neither smokers nor drinkers. There is need to examine the separate contribution of non-smoking drinkers, non-drinking smokers and smoking drinkers to the measured inequality in health. In Table 5, we report the combined percentage contribution of smoking and alcohol use to income-related health inequality. The analysis is limited to self-reported health and the health index. The combined use of cigarettes and alcohol accounts for 9.83 to 17.61% of all measured inequality in the health index in Wave 1 and Wave 4, respectively, with a maximum of 2.8% for self-reported health (see Wave 1 of Table 5). The contributions from non-smoking drinkers are generally higher than those from non-drinking smokers. This suggests that individuals who are both smokers and drinkers have higher risk of ill-health than non-smoking drinkers and non-drinking smokers. Because the effects of smoking on health are not immediate, we also examine the contribution of smoking duration on health separately. For smoking-related ill-health, the estimates are higher when smoking duration is considered than when smoking participation is used (see Table 6). In the analysis, we control for covariates that are associated with health, so that the estimated effects of smoking and alcohol consumption are unconditional.

**Table 5:** The Combine contribution of Smoking and alcohol to income-related inequalities in health by wave

	Wave 1						Wave 2					
	Health Index			Self-reported health			Health Index			Self-reported health		
	Elasticity	Contribution	%	Elasticity	Contribution	%	Elasticity	Contribution	%	Elasticity	Contribution	%
Individual only smokes	0.004	0.000	1.521	0.001	0.000	0.490	0.008	0.000	3.125	0.000	0.000	0.127
Individual only drinks	0.021	0.007	8.564	0.003	0.001	1.049	0.034	0.011	13.527	0.002	0.001	0.775
Individual drinks and smokes	0.025	0.018	9.834	0.007	0.005	2.802	0.034	0.025	13.533	0.003	0.001	1.046
	Wave 3						Wave 4					
	Health Index			Self-reported health			Health Index			Self-reported health		
	Elasticity	Contribution	%	Elasticity	Contribution	%	Elasticity	Contribution	%	Elasticity	Contribution	%
Individual only smokes	0.022	0.001	8.879	0.002	0.001	0.835	0.003	0.000	1.205	0.000	0.000	0.004
Individual only drinks	0.024	0.013	9.639	0.003	0.000	1.183	0.042	0.009	16.786	0.001	0.001	0.322
Individual drinks and smokes	0.042	0.010	16.962	0.006	0.001	2.263	0.044	0.025	17.613	0.006	0.001	2.569

Notes: Results presented in this table are elasticities, contributions, and percentage contributions of cigarette smoking and alcohol consumption to income-related health inequality. The results are obtained by decomposing the income-related health inequality indices into health related covariates, including smoking and alcohol use. Self-reported health is binary and the health index is continuous with high values representing poor health outcomes. The tobacco and alcohol use variables is categorical. Other covariates include household per capita income, gender, categories for age, province of residence, race, marital status, and education

**Table 6:** The contribution of smoking duration on income-related inequalities by wave

	Wave 1			Wave 2		
Variable	Elasticity	Contribution	%	Elasticity	Contribution	%
Self-reported health	0.002	0.014	0.614	0.001	0.009	0.353
Health Index	0.013	0.114	5.126	0.019	0.193	7.659
	Wave 3			Wave 4		
Variable	Elasticity	Contribution	%	Elasticity	Contribution	%
Self-reported health	0.004	0.038	1.720	0.004	0.003	1.779
Health Index	0.035	0.315	14.104	0.037	0.025	14.674

Notes: Results presented in this table are elasticities, contributions, and percentage contributions of smoking duration to income-related health inequality. The results are obtained by decomposing the income-related health inequality indices into health related covariates, including smoking duration. Self-reported healthis binary while the health index is continuous with high values representing poor health outcomes. Smoking duration is continuous ranging from zero. Other covariates include household per capita income, gender, categories for age, province of residence, race, marital status, and education

## Discussion

International evidence has attempted to measure the contribution of smoking, alcohol use and obesity to socioeconomic-related inequalities in self-reported health [4, 44, 45]. A consistent positive contribution of these lifestyle factors to income-related inequality in self-reported health has been identified. In the context of South Africa, a number of studies have highlighted the prevalence of both communicable and non-communicable diseases among poor individuals and households (see [1, 2]). These studies show inequality in the distribution of health outcomes between the poor and the rich, but do not show how this can be attributed to differences in lifestyles. This paper attempts to provide evidence of the contribution of smoking and alcohol use on income-related health inequality in South Africa and in the process contribute to the international literature by incorporating more objective measures of lifestyle-related health outcomes.

We first measure smoking-related health inequality and income-related health inequality for a number of health indicators, using NIDS 2008–2015, a national representative panel data set for South Africa. Second, we decompose the income-related health inequality indices into observable health-related covariates, including smoking and alcohol consumption. The findings suggest that, for all health indicators, the burden of ill-health is significantly concentrated among individuals with high smoking intensity and longer smoking duration. The magnitude of the inequalities varies significantly among diseases and across waves. The majority of the health indicators show marked inequality related to smoking duration rather than to smoking intensity. For example, the concentration indices for the health index are larger when smoking duration is used than when smoking intensity is used. Effective tobacco control policies that reduce the prevalence of tobacco use among the poor than the rich are likely to narrow smoking-related health inequalities.

Two different measures of income are used to compute income-related inequality, namely, household per capita income and household per capita income by adult equivalent scale. Inequality in the distribution of most of the diseases is concentrated among the poor than among the rich. The findings are generally consistent across the different measures of income. The magnitude of income-related inequality varies across waves (it increases as we move from Wave 1 to Wave 4) and among health indicators. Using repeated cross-sectional data to explore inequality trends in the United Kingdom (UK), [25, 45] confirm a modest increase in income-related health inequality over time. This paper has shown that smoking and alcohol consumption contribute positively to income-related inequality in health, deteriorate health significantly, and are concentrated mainly among the poor (*CCIs >* 0). In general, the contributions of alcohol consumption are larger than the contributions from cigarette smoking (probably due to the proportion of alcohol users in the sample) and both increase over time. In all waves, the contributions of smoking and alcohol use to income-related inequality are significant, but less than the overall contributions from other factors.

Therefore, reducing tobacco consumption and alcohol use could be an effective policy for reducing the prevalence of lifestyle-related diseases and narrowing inequalities in health in South Africa. While the prevalence of these avoidable risky health behaviours are expected to be concentrated among the poor, related interventions are also more likely to cause the poor to quit or not to initiate such behaviours. However, the poor are more likely than the rich to switch to consuming illicit cigarettes and alcohol (cheaper options), but these unregulated alcohol products may be more dangerous to health. To validate this argument, information on consumption of illicit alcohol and tobacco products at an individual level is required. Unfortunately, data on illicit consumption of both tobacco and alcohol is difficult to collect and rarely collected in surveys.

The existing data allows us to analyse smoking-related inequality in health. However, it could not be used to analyse alcohol-related inequality in health. NIDS has no information on the intensity and duration of alcohol consumption, limiting the discussion on alcohol-related health inequality. A comparison of smoking-related inequality in health and alcohol-related inequality in health in South Africa requires a comprehensive data set on alcohol consumption in the country.

The lifestyle-related diseases profile used are self-reported, but are based on medical diagnoses and are regarded as objective indicators of health. However, the assumption is that respondents fully understand and accurately report their diagnoses. It seems plausible that higher income groups or the more educated have a better understanding of medical terminology and more accurately report their health problems. In addition, those who have limited access to medical care will not know of their disease profile (those with less income and limited access to quality medical care may have these diseases but may not be aware of them). If this is the case, measurement error may bias our findings on the extent of health inequality. To address this bias requires individuals to be diagnosed of these diseases at the point of data collection.

## Conclusion

This paper contributes to existing evidence by incorporating more objective measures of health that are directly associated with smoking and harmful alcohol use. Inequality in the distribution of most of the associated diseases is concentrated among the poor than among the rich, irrespective of the income measure used. Evidence suggest that smoking and harmful alcohol use contribute positively to income-related inequality in health. The contributions from alcohol are generally larger (probably due to the proportion of alcohol users in the sample). For all health indicators, the burden of ill-health is significantly higher when more objective measures of health are considered, than when self-reported health is considered. The contributions of smoking and alcohol use to income-related inequality are significant but less than the overall contributions from other factors. Reducing tobacco and alcohol use could be an effective strategy for reducing the prevalence of such lifestyle-related diseases, and narrowing inequalities in health. While the prevalence of these avoidable risky health behaviours are expected to be concentrated among the poor, related interventions are more likely to cause them to quit or not to initiate such behaviours. Concerted efforts of the government to effectively reduce the rate of tobacco and harmful alcohol use, especially among the poor are likely to narrow health inequalities in South Africa.

## Abbreviations

CCI: Corrected concentration index
CI: Concentration index
NCDs: Non-communicable diseases
NIDS: National income dynamic survey
SRH: Self-reported Health
UK: United Kingdom
WHO: World Health Organisation

## Footnotes

1 The EQ-5D is a generic measure of health status which is applicable to a wide range of health conditions and treatments, and provides a descriptive profile that is reducible to a single index value for health status.
